# Structural Integrity of the Uncinate Fasciculus and Resting State Functional Connectivity of the Ventral Prefrontal Cortex in Late Life Depression

**DOI:** 10.1371/journal.pone.0022697

**Published:** 2011-07-22

**Authors:** David C. Steffens, Warren D. Taylor, Kevin L. Denny, Sara R. Bergman, Lihong Wang

**Affiliations:** Department of Psychiatry and Behavioral Sciences, Duke University Medical Center, Durham, North Carolina, United States of America; University of Illinois at Chicago, United States of America

## Abstract

**Background:**

Neuroimaging studies in late life depression have reported decreased structural integrity of white matter tracts in the prefrontal cortex. Functional studies have identified changes in functional connectivity among several key areas involved in mood regulation. Few studies have combined structural and functional imaging. In this study we sought to examine the relationship between the uncinate fasciculus, a key fronto-temporal tract and resting state functional connectivity between the ventral prefrontal cortex ((PFC) and limbic and striatal areas.

**Methods:**

The sample consisted of 24 older patients remitted from unipolar major depression. Each participant had a magnetic resonance imaging brain scan using standardized protocols to obtain both diffusion tensor imaging and resting state functional connectivity data. Our statistical approach compared structural integrity of the uncinate fasciculus and functional connectivity data.

**Results:**

We found positive correlations between left uncinate fasciculus (UF) fractional anisotropy (FA) and resting state functional connectivity (rsFC) between the left ventrolateral PFC and left amygdala and between the left ventrolateral PFC and the left hippocampus. In addition, we found a significant negative correlation between left ventromedial PFC-caudate rsFC and left UF FA. The right UF FA did not correlate with any of the seed region based connectivity.

**Conclusions:**

These results support the notion that resting state functional connectivity reflects structural integrity, since the ventral PFC is structurally connected to temporal regions by the UF. Future studies should include larger samples of patients and healthy comparison subjects in which both resting state and task-based functional connectivity are examined.

## Introduction

Studies over the past decade have improved our understanding of the neural circuits underlying mood and the development of depression. Projections from the prefrontal cortex (PFC) to the limbic areas (amygdala, hippocampus) and brainstem nuclei modulate endocrine, autonomic, and behavioral aspects of emotion [1]. Phillips et al. [2] presented models of emotion regulation in mood disorders that implicate the lateral PFC (which operates by a feedback mechanism) and the medial PFC (which operates by a feedforward mechanism) in regulation of emotion perceptual processes in amygdala and ventral striatum.

Two neuroimaging modalities, diffusion tensor imaging (DTI) and functional magnetic resonance imaging (fMRI), are used to examine, respectively, structural integrity and functional connectivity of brain regions. While previous fMRI studies have focused largely on activation or deactivation of key regions in emotion regulation circuits, recently attention has also been given to connectivity or synchronization of neural oscillations among these regions. Combining structural findings using DTI and functional connectivity using fMRI has great mechanistic appeal and has been used to examine performance on cognitive tasks in normal volunteers [3], [4] and in pathological states such as traumatic brain injury [5], schizophrenia [6], temporal lobe epilepsy [7] and opioid dependence [8]. There are no current published studies combining DTI and fMRI in late life depression (LLD), although one recent study examining key brain regions involved in mood and mood disorders has concluded that functional connectivity reflects structural connectivity in these areas [3]. We sought to use both DTI and fMRI to examine the relationship between structural integrity of PFC white matter and functional connectivity between frontal and limbic regions among older depressed patients.

In LLD, structural disruption in white matter connecting key brain areas has been proposed as a pathological mechanism. We have suggested that LLD involves a disconnection syndrome affecting frontostriatal [9] and fronto-limbic [10] areas. One candidate connecting brain structure that may be associated with depression is the uncinate fasciculus (UF), a prominent white matter tract connecting inferior frontal and temporal lobe regions, including amygdala and hippocampus [11]. Sheline reported greater white matter lesion volume in left UF among older depressives than older controls, and that white matter lesion volume in left UF correlated with performance on executive function tasks [12]. However, it is unclear whether relationship between the structural measure of left UF white matter lesion burden and executive dysfunction was mediated by corresponding alterations of functional connectivity between the prefrontal and limbic striatal regions. To address the question of whether structural integrity and functional connectivity are related, we sought to investigate the relationship between the UF white matter integrity and resting state functional connectivity in regions connected by the UF. Following the results of a previous study in young normal control subjects [3], we hypothesized that higher fractional anisotropy (FA, a measure of structural integrity) on DTI (i.e., greater structural integrity) would be positively associated with functional connectivity between medial prefrontal and temporal regions. In addition, we explored whether there would be a relationship between structural integrity of the UF and broader functional connections of the medial prefrontal cortex.

## Methods

### Ethics statement

The study was approved by the Institutional Review Board at Duke University Medical Center. After complete description of the study to the subjects, written informed consent was obtained.

### Subjects

The sample consisted of depressed subjects enrolled in the NIMH supported Conte Center for the Neuroscience of Depression in the Elderly and the Neurocognitive Outcomes of Depression in the Elderly (NCODE) study at Duke University Medical Center. At study entry, depressed subjects met criteria for a current episode of unipolar major depression and were at least 60 and older. Exclusion criteria included presence of another major psychiatric illness such as schizophrenia, schizoaffective disorder, bipolar disorder, lifetime alcohol or substance dependence, and dementia. Patients with psychotic depression were included, as were those with comorbid anxiety disorders, as long as major depression was deemed by the study psychiatrist to be the primary psychiatric disorder. Aside from dementia, other neurological illnesses that could affect structural brain MRI scans were excluded, such as Parkinson's disease, multiple sclerosis, and seizure disorder. Subjects with contraindications to brain MRI were also excluded. After complete description of the study to the subjects, written informed consent was obtained. A full description of assessments for the study has been described previously [13].

For the present study, we recruited patients who were in a remitted or a partially remitted state from major depression with a Montgomery-Åsberg Depression Rating Scale (MADRS) [14] score ≤15 at the time they participated in the MRI study and who reported low symptoms for at least the three prior months. Prior to the fMRI, subjects' cognitive function was assessed using a short, 30-minute battery of neuropsychological tests. The neuropsychological tests include: Mini-Mental State Exam, Category Fluency, Hopkins Verbal Learning Test-Revised (HVLT-R), Immediate and Delayed Story Recall from the Rivermead Behavioral Memory Test, WAIS-III Digital symbol, WAIS-III Digit span, Trail Making Test (Trail A and Trail B), and Stroop Color and Word Test. The battery has extensive psychometrics to support its reliability and validity in detecting dementia.

### Image acquisition

Subjects were imaged with a 3.0 Tesla whole-body MRI system (3.0 T GE EXCITE HD scanner (GE Medical Systems, Milwaukee, Wisconsin) using the 8-channel head coil. A custom-built head holder was used to prevent head movement. Oblique spoiled gradient-recalled acquisition images (three-dimensional, whole-brain) were acquired parallel to the anterior commissure (AC) - posterior commissure (PC) plane for high-resolution T1-weighted structural images with a matrix of 256×256×169, slice thickness of 1 mm. We first acquired five-minute resting-state BOLD images using SENSE inward spiral sequence with the following parameters: TR = 2000 ms, TE = 31 ms, FOV = 24 cm, flip angle = 90°, matrix = 64×64×34, slice thickness = 3.75 mm with 3.75 mm3 isotropic voxels. Following the resting-state bold imaging, we collected DTI images using SENSE acquisition. The DTI parameters were as follow: 256×256×72 matrix with 1×1×2 mm^3^ resolution, TR = 1700 ms, TE = 7.5 ms, flip angle = 90°, 25 non-collinear encoding directions, b factor of 1000s/mm^2^, 8-channel EPI acquisition, 7 min total imaging time.

### Uncinate region-of-interest measurement

For image pre-processing, registration, and normalization, we used Tract-Based Spatial Statistics (TBSS, http://www.fmrib.ox.ac.uk/fsl/tbss/index.html), part of the FSL analysis package (FMRIB's Software Library, www.fmrib.ox.ac.uk/fsl). Briefly, each subject's original data were corrected for the effects of head movement and eddy currents, and then every FA image was aligned to every other one to identify the “most representative” one, and which as used as the target image. This target image was then affine-aligned into MNI152 standard space, and every image was transformed into 1×1×1 mm MNI152 space by combining the nonlinear transform to the target FA image with the affine transform from that target to the MNI152 standard space. The nonlinear transformation using the most representative image from our sample allowed us to better align and normalize individual images than simply using the standard image as a target image.

Left and right UF structural regions of interest (ROIs) were created from the JHU-DTI-81 white-matter labeled atlas, which was coregistered with the MNI152 standard space. The mean FA value within the left and right UF for each subject was computed for further regression analysis with the BOLD resting-state functional connectivity.

### Resting-State Functional Connectivity (rsFC) measurement

The resting-state BOLD contrast images were pre-processed using FEAT (FMRI Expert Analysis Tool) Version 5.98, part of the FSL analysis package, through standard image pre-processing procedures including slice-timing alignment, motion correction, coregistration, non-brain voxels extraction, normalization (non-linear transformation), and smoothness (6 mm3 kernel). Temporal filtering settings were applied using a high-pass filter (Gaussian-weighted least-squares straight line fitting, with sigma = 100.0 s) and a low-pass filter (Gaussian low-pass temporal filtering: HWHM 2.8 s) following Biswal and colleagues [15].

We carried out seed-based functional analyses by extracting the BOLD time series from ROIs. Given our particular interest in regions that are connected through the uncinate fasciculus, we chose the following ROIs as seed regions (co-ordinates for the center of the ROI): the amygdala (left, −19, −4, −18, right, 27, −3, −15), hippocampus head (HChead, left, −20, −5, −23, right, 26, −7, −20), hippocampus tail (HCtail, left, −23, −25, −12, right, 23, −25, −12), parahippocampus (PHC, left, −25, −39, −10, right, 25, −39, −10), subgenual cingulate (Cg25,4, 22, −8), ventromedial prefrontal cortex (vmPFC, left, −6, 50, −9, right, 7,54, −9), lateral orbitofrontal cortex (OFC, left, −31, 42, −8, right, 31, 42, −8), and ventrolateral prefrontal cortex(vlPFC, left, −47, 26, 15, right 47, 26, 15). The center coordinates for these regions were determined based on the peak activations evoked by an emotional memory task (not included in this report) along with a meta-analysis study [16], which documented the most frequently reported regions in studies with major depression. The ROIs were 5 mm radius spheres from the center drawn using the MRIcron (http://www.nitrc.org/projects/mricron/) software, which allowed us to set constrains of the ROIs not exceeding a structural boundary. The timecourse of each ROI was then entered as a regressor into the first-level (within- subject) general linear model (GLM) using FEAT. Nuisance regressors (global signal, white matter, cerebrospinal fluid, and motion parameters) were also entered into the model.

### Statistical Analyses

Initially, we examined the relationship between the left and right UF FA values and depression symptom severity using Pearson correlations. A Pearson r greater than 0.4 and p value less than 0.05 were considered significant for the ROI analyses.

To examine the relationship between the functional connectivity and individual variation in FA value of the left and right UF, the demeaned UF FA value (raw value minus mean value) for each subject was entered as a regressor into the third level general linear model (GLM) with the left and right UF in separate models. We also used age as a nuisance regressor to remove age effect in the third level analyses. Statistical results used a voxel significance threshold of z>2.3 and a whole-brain-corrected cluster-significance threshold of p<0.05. Significant clusters were selected as ROIs, and the plots of mean rsFC value within each ROI against the left and right UF FA value were used to double confirm the voxel-based whole-brain analysis.

## Results

There were 24 subjects in the sample, including 12 women and 12 men. As shown in Table 1, the sample had a mean age of 69.5 and mean educational level of 15. The mean MADRS score of 3.9 reflects the remitted state of participants, and the mean MMSE of 28.9 shows that they were generally cognitively intact (Table 1). The mean FA value for the left UF was 0.34 (0.28) and for the right UF was 0.42 (0.11). Performance on the memory and executive function-related tests from the neuropsychological battery are also listed in Table 1. Of note, the FA value for the left UF was not correlated with either depression severity or duration of depressive disorder (results not shown).

**Table 1:** Sociodemographic and Clinical Characteristics of Participants.

	Patients N = 24
Age, mean (SD)	69.5 (6.7)
Sex, Number of Females/Males	12/12
Education, mean (SD), years	15.0 (2.9)
Age of depression onset, mean (SD)50 years or less, NOver 50 years, N	44.0 (18.2) 11 13
Multiple episodes, N	17
*Number of depressive episodes, mean (SD)	4.32 (4.4)
Montgomery Asberg Depression Rating Scale (MADRS) score Mean (SD)0–7, N8–15, N	3.9 (3.9) 19 5
MMSE, mean (SD)	28.9 (1.4)
Medicated with antidepressants, N	12
Memory	
Rivermead Behavioral Memory Test - delay, mean score (SD)	7.0 (3.5)
Hopkins Verbal Learning Test - delay, mean score (SD)	8.3 (3.6)
Executive function	
Digit span, mean score (SD)	13.5 (3.7)
Stroop Color and Word Test, mean score (SD)	32.2 (10.6)

*Data are missing on four subjects who could not provide number of episodes.

### Resting-state functional connectivity (rsFC)

The rsFC map from each seed region is shown in supplementary Figure S1. Briefly, the amygdala showed connectivity with the OFC, bilateral hippocampus, insula, ventral putamen, and middle temporal cortex. The hippocampus tail showed connectivity with the vmPFC, Cg25, bilateral amygdala, middle temporal cortex and temporal pole, fusiform gyrus, thalamus, dorsal brainstem, insula and cerebellum. The Cg25 seed was functionally connected with the vmPFC, bilateral amygdala, hippocampus, caudate, putamen, superior temporal sulcus (STS), and fusiform gyrus and visual cortex. The lateral OFC was functionally connected with the ventrolateral prefrontal cortex (vlPFC), caudate, insula, and thalamus. The vlPFC seed showed connectivity in the dACC, inferior parietal cortex (IPC), STS, and the posterior cingulate cortex (PCC). The vmPFC seed showed connectivity in the default-mode network system: the dorsomedial prefrontal cortex (dmPFC), the PCC, and bilateral IPC.

### Correlation of FA in the left UF with rsFC

**Figure 1:**
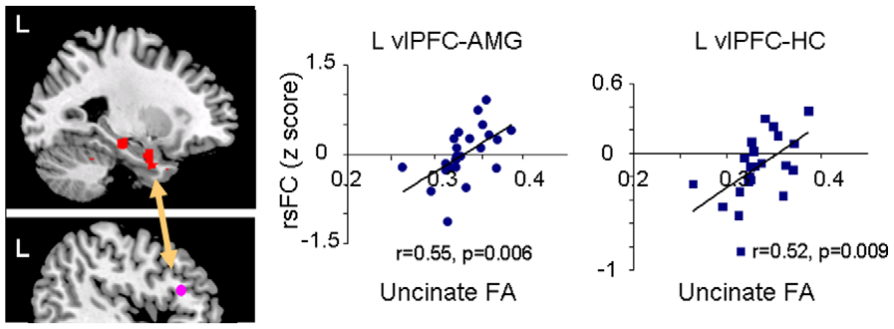
A positive correlation between the uncinate fasciculus integrity and functional connectivity using a seed in the left ventrolateral prefrontal cortex (vlPFC) region. Left, brain image illustrating brain regions which showed the correlation of left uncinate integrity with the left vlPFC-amygdala connectivity. Right, the plots of the significant clusters in the amygdala and hippocampus that showed significant functional connectivity with the vlPFC seed.

**Figure 2:**
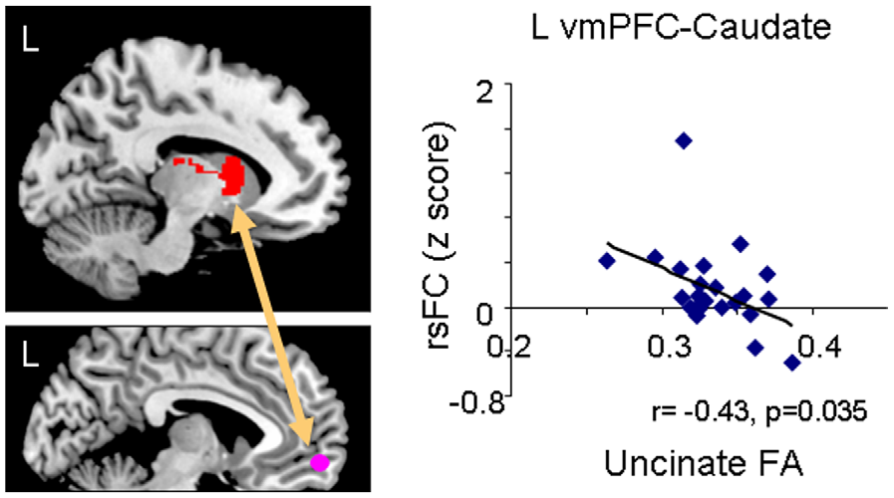
A negative correlation between the uncinate fasciculus integrity and functional connectivity using a seed in the left ventrolateral prefrontal cortex (vlPFC) region. Left, brain image illustrating brain regions which showed the correlation of left uncinate integrity with the left vlPFC-caudate connectivity. Right, the plots of the significant clusters in the caudate that showed significant functional connectivity with the vlPFC seed.

Using the left UF FA value of each subject as a regressor, we conducted whole-brain voxel-based regression analyses to investigate the relationship of the left UF integrity with the rsFC from each seed region. In whole brain analysis on the left vlPFC seed rsFC map, we found positive correlations between left vlPFC-amygdala rsFC and left UF FA, and between the left vlPFC-hippocampus rsFC and left UF FA. Using the significant clusters as amygdala and hippocampus ROIs, we confirmed using ROI analyses the correlation of the vlPFC-amygdala rsFC and left UF FA (r_23_ = 0.55, p = 0.006) and the correlation of vlPFC-hippocampus rsFC and left UF FA (r_23_ = 0.52, p = 0.009) (see Figure 1 and Table 2). In addition, in whole brain analysis on the vmPFC seed rsFC map, we found a significant negative correlation between left vmPFC-caudate rsFC and left UF FA (see Figure 2 and Table 2). The ROI analysis on the significant cluster of the caudate also confirmed the voxel-based analysis (r_23_ = −0.43, p = 0.035).

**Table 2:** Prefrontal cortex (PFC) regions for which their resting-state functional connectivity (rsFC) with limbic striatum regions was significantly correlated with uncinate fasciculus fractional anisotropy value.

Seed Regions	Regions of significant clusters	z score	X_MNI_	Y_MNI_	Z_MNI_
Positive Correlation					
Left ventrolateral PFC	Left Amygdala	3.57	−24	−3	−22
	Left Hippocampus	4.04	−38	−22	−14
	Right Hippocampus		24	−28	−8
Negative Correlation					
Left ventromedial PFC	Left Caudate	3.7	−10	10	8
	Left Thalamus	3.67	−16	−22	16

Whole-brain analyses failed to show a significant correlation of vmPFC-amygdala or vmPFC-hippocampus rsFC with left UF FA either using the vmPFC as a seed or using the left amygdala or HCtail or HChead as a seed. However, using the above significant amygdala and hippocampus clusters found in the vlPFC seed analysis as ROIs to do a ROI analysis, the results showed that vmPFC-HC rsFC was positively correlated with the UF FA (r_23_ = 0.46, p = 0.02).

The right UF FA did not correlate with any of the seed region based connectivity.

## Discussion

Our major finding is that left UF FA values were positively correlated with rsFC between left vlPFC and both the amygdala and the hippocampus. Since the ventral PFC is structurally connected to temporal regions by the UF, these results support the notion that resting state functional connectivity reflects structural integrity. Our findings are consistent with a previous report in healthy younger adults examining the constituent structures of the default mode network [3]. We did not include non-depressed older controls in this study, so we can only propose that, similar to what we might find in non-depressed elderly, the finding of a relationship between positive FA and rsFC reflects an intact structural connection. Alternatively, since we studied the relationship between white matter integrity and rsFC in depressed patients only, our results might be specific to this older depressed group. Depression has been associated with loss of normal top-down control between the prefrontal cortex and temporal regions [11], assuming an otherwise intact neural communication system. If our results are specific to depressed patients, loss of top-down control might be reflected in higher rsFC among patients with greater structural integrity (higher FA) of the UF. Among those with lower FA, we can infer impaired communication between the two regions may lead to poor connectivity, i.e., a z scored rsFC approaching zero. We found that most of the lower FA values are associated with rsFC values near zero (Figure 1). It is not clear why some rsFC values are less than zero. Thus, our results indicate the need to further investigate the relationship between the white matter integrity and rsFC in both healthy and patient groups.

We also found a negative correlation between the UF FA value and degree of connectivity of the vmPFC and caudate. This is interesting, given that the vmPFC and caudate are structurally connected via corticostriatal fibers rather than through the UF. Whether the negative correlation between UF FA and vmPFC-caudate resting state connectivity involves a compensatory means of exerting top-down control through corticostriatal fiber connections in the face of poor UF structural integrity is not clear. Such a compensatory mechanism is often seen in task-related fMRI studies where subjects engage in cognitively demanding tasks, and it might also be seen in resting state in case the compensatory pathways are frequently triggered by habitual thoughts.

Activation in both the vlPFC and vmPFC has been reported in emotion regulation [2], [17], [18], [19]. Increased activation in the vlPFC, dlPFC and vmPFC, and decreased activation in the OFC and amygdala were found in healthy individuals when they were reappraising stimuli to reduce negative affect [18], [19], [20]. The PFC projections to the amygdala are thought to exert a top-down, inhibitory influence. The vlPFC/dlPFC-amygdala inhibitory effect is thought to be exerted via the vmPFC, which has direct connections with both vlPFC/dlPFC and also with the amygdala [18], [21]. Both vlPFC and vmPFC negatively correlated with the activation of the amygdala in healthy subjects during reappraisal compared with simply attending to negative stimuli; however, the mediation effect of vmPFC was not seen in depression [18]. On the other hand, one study on the role of vlPFC in reappraisal found that the vlPFC-nucleus accumbens/ventral striatum pathway predicted reappraisal success and vlPFC-amygdala pathway predicted reappraisal failure [22]. The association of vlPFC-amygdala rsFC with UF FA we found in depressed patients may provide an anatomical support for the reappraisal failure which is the core feature of cognitive bias in depression. Future studies comparing the relationship of UF FA with the vlPFC-amygdala connectivity during resting state and during emotional regulation tasks between LLD and healthy subjects will help to further understand the positive findings here.

Also worth noting is the laterality of our findings. All the significant correlations were seen in the left UF, but not the right. This is consistent with our previous report of lower FA in the left UF among older adults with early onset depression compared with mid- and late-onset older depressives or nondepressed subjects, while analyses of the right UF were not statistically significant [23]. Sheline et al. reported greater white matter lesion volume in left UF among elderly depressives than elderly controls, and that among depressives, white matter lesion volume in left UF correlated with performance on tasks of executive function [12]. However, one must be cautious about inferring that left-sides findings are necessarily pathological merely because of a lack of findings on the right side. As stated above, it is critical to further examine whether our findings are specific to depressed patients. Moreover, others have noted findings for the right UF among patients with bipolar disorder [24].

In sum, our results represent an examination of the connectivity of the brain at rest in the context of geriatric depression. It is thus not surprising that our findings differ from other studies that involve task-related activation paradigms. Lack of a healthy older comparison group limits our ability to draw conclusions about the specificity of our findings in geriatric depression. Future studies should include larger samples of patients and healthy comparison subjects in which both resting state and task-based functional connectivity are examined. In addition, future research should examine the structural integrity of other white matter tracts, including the corticostriatal fibers that connect the prefrontal cortex and the basal ganglia, which would further enhance our understanding of the neural mechanisms underlying late-life depression.

## Supporting Information

Figure S1 **Resting state functional connectivity (rsFC) map of six seed brain regions that were examined.** The regions were amygdala, hippocampus, cingulum area 25 (Cg25), orbitofrontal cortex (OFC), ventromedial prefrontal cortex (vmPFC) and ventrolateral prefrontal cortex (vlPFC).
(TIF)
