# Supplementary figures and images for: Structural Integrity of the Uncinate Fasciculus and Resting State Functional Connectivity of the Ventral Prefrontal Cortex in Late Life Depression
Source: PLoS One. 2011 Jul 22;6(7):e22697. doi: 10.1371/journal.pone.0022697 (PMC3142185; doi:10.1371/journal.pone.0022697)

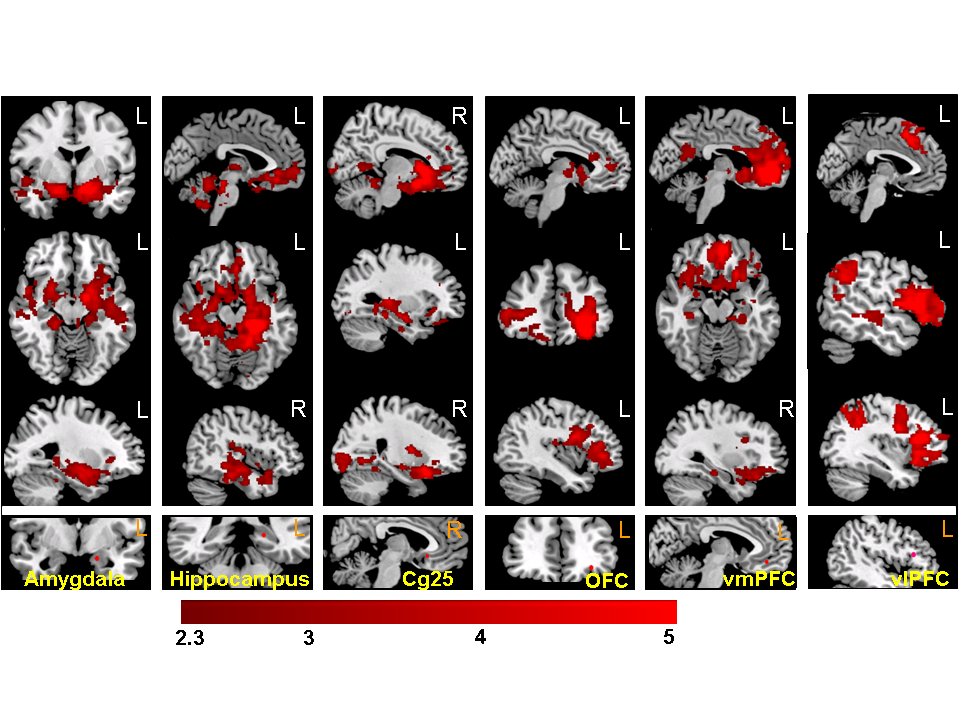

Supplement: Figure S1 — Resting state functional connectivity (rsFC) map of six seed brain regions that were examined. The regions were amygdala, hippocampus, cingulum area 25 (Cg25), orbitofrontal cortex (OFC), ventromedial prefrontal cortex (vmPFC) and ventrolateral prefrontal cortex (vlPFC). (TIF) [file pone.0022697.s001.tif]
